# Life design facing the fertility gap: promoting gender equity to give women and men the freedom of a mindful life planning

**DOI:** 10.3389/fpsyg.2023.1176663

**Published:** 2023-05-31

**Authors:** Elisabetta Camussi, Daria Meneghetti, Riccardo Rella, Maria Luisa Sbarra, Elena Calegari, Cinzia Sassi, Chiara Annovazzi

**Affiliations:** ^1^Department of Psychology, University of Milan-Bicocca, Milano, Lombardy, Italy; ^2^Department of Human and Social Sciences, University of Valle D’Aosta, Aosta, Aosta Valley, Italy

**Keywords:** fertility gap, gender equality, life design, Agenda 2030, social sustainability

## Abstract

Nowadays, society is characterized by enormous and rapid changes, erratic careers, gender discrimination, injustices, and inequities. Discrimination includes professional and educational segregation, the gender pay gap, stereotypical gender roles, and social expectations. In this context, phenomena called low fertility and fertility gap are increasing. Indeed, the birth rate necessary to ensure the replacement of the population is not reached, with severe repercussions at a social, environmental, and economic level. This study aimed to investigate 835 women’s perceptions of the desire for motherhood and the associated difficulties. Hierarchical multiple regression and thematic decomposition analyses first highlight a significant difference between the number of children women realistically plan and the ideal number of children they would like. Secondly, the results showed how the parenthood choice is connected to the perception of social and gender inequity. Finally, in a Life Design perspective, preventive actions will be described to support women to get back to the center of life choices, building dignified fair paths and family projects.

## 1. Introduction: the social-economic context

A profound economic, health, and social crisis significantly impacted the labor market in the last decade. Italy fell into a robust recession: Italy’s GDP for 2020 was $1,892.57B, a 5.9% decline from 2019; in 2019, it was $2,011.29B, a 3.86% decline from 2018, in which GDP was $2,091.93B, increased by 6.63% compared to 2017 (The World Bank, IBRID – IDA, 2023). During these years and in the last two decades, the current working context started to be qualified by a different organization of work and a high level of uncertainty. Fewer stable job positions corresponded to a higher number of flexible, temporary, and precarious jobs (Rubery and Piasna, 2017), with temporary, part-time, or casual (so-called “zero-hours”) contracts. Workers must frequently face different professional transitions (Savickas et al., 2009). Due to this marked economic recession in Western society, job losses increased drastically, especially for women and youth, influencing their ability to design their professional and personal lives. Nowadays, the number of women in the occupational field is inadequate. In 2022, for most countries, women marked a higher unemployment rate than men: the European unemployment rate for women was 6.4%, higher than the rate for men, which was 5.8%. In 2021 in Italy, the women’s unemployment rate was 11.26%. Besides, almost half of the employed women in the EU in 2022 were working part-time (47%), nearly two times the rate for men (26%) (OECD, 2021; Eurostat, 2023). Given the higher employment rates of women in more vulnerable contract forms, women experienced lower forms of social protection, such as maternity benefits and unemployment benefits, and lower pensions (International Labour Organization, 2017). The characteristics of the current labor market, high unemployment rates, and extreme precariousness could be considered professional barriers that hinder the possibility of achieving professional, personal, and life aims (Swanson and D’Achiardi, 2005). These barriers and the considerable uncertainty facilitate in people the idea that the future will be unstable and economically unsatisfactory without the possibility of planning personal growth. Indeed, the increase in the unemployment rate reduces the chance of personal development and achievement of one’s aspirations, particularly for youth and women (Evans and Gibb, 2009). For this reason, youth and women consider their professional life related to a higher level of vulnerability, which makes it difficult for them to find meaning in work. In addition, despite several laws that support the female presence in the labor market, women must face a series of external barriers (e.g., discrimination in the work context, gender stereotypes, etc.) and internal ones (e.g., the managing of family work and multi-role conflict, etc.) (Swanson and D’Achiardi, 2005; Betz, 2006). Considering the external barriers, women undergo discrimination, such as the gender pay gap (Pascall, 2008) and inequity in promotions. On average, working women in the EU earn 13% less per hour than men – 4.2% in Italy - although they have the same competence as male colleagues (Eurostat, 2020). In the world of work, men tend to choose other men for responsibility roles and higher positions, slowing the progress of women (Lundberg and Stearns, 2019). Indeed, there is a form of vertical segregation – the glass ceiling - a phenomenon for which it is complicated for women to reach the top positions (Ryan and Haslam, 2005). For example, in 2021, only 30.6% of board members in the EU’s most prominent companies were women (European Institute for Gender Equality (EIGE), 2022). This phenomenon is explained by some co-optation processes, such as using different judgments because of gender belonging: women are often considered less competent than men (Gilrane et al., 2019). The interiorization of this process negatively influences women’s perception of being able to build a career or achieve professional goals. Therefore, women tend to review their professional projects with some stopgap professional activities or an early exit from work for inactivity (Van der Lippe and Lippènyi, 2020). Among the external professional barriers, it is also possible to include occupational gender stereotypes that lead to expectations about professional roles that men and women should assume because they are biologically male and female (Ellemers, 2018). Women are described as warm and caring - traits associated with loving, educational, and nursing jobs - while men are defined as competent and determined, ideal for leadership positions (Haines and Stroessner, 2019). These stereotyped beliefs penalize women, limiting their access to traditionally male occupations with greater social prestige, remuneration, and career prospects (OECD, 2021). Indeed, these stereotypes accentuate the association between specific professional categories and gender belonging, influencing the choice of an appropriate path for each gender (Gysbers et al., 2009). All the external barriers have an impact on the internal ones. For instance, tracing back to the stereotypes associated with genders, women are considered primarily responsible for caring for the home and family (Camussi et al., 2021). Most women are socialized from an early age to be wives and mothers, accountable for the care of the house and devoted to their offspring, thus causing female workers to experience more significant work-home conflict (Piccitto, 2018). The difficulty of reconciling work and family responsibilities – one of the internal barriers - constitutes a substantial obstacle to gender equity (Suk, 2010). It negatively affects women’s personal and professional well-being and their freedom to design and build their future careers and life (Kulik et al., 2016). Although the differences between partners in the domestic workload have diminished in recent years, a significant gender gap continues to exist. Indeed, it is possible to talk about the “triple presence of women”: employed in their career (first), occupied with taking care of the home (second), and caring for children and elderly parents (third). All these barriers put women in the condition to make a decisive choice between their family aspirations and careers. On the one hand, motherhood often pushes women to change jobs, decrease workplace hours, or give up their working life because of increased stress and emotional exhaustion (Burke et al., 2012). On the other hand, women who struggle with responsibilities and work commitments can reconsider own and couples’ reproductive choices.

## 2. Low fertility and fertility gap in Italy

According to the most recent data ISTAT (2019), Italy is living through a new demographic decline started in 2015. For instance, on the 31st of December 2019, with a population of 60,244,639, of which 8.8% were foreigners, the resident population was almost 189 thousand units lower (188,721) compared to the beginning of the year. Indeed, 2019 was one of the years with the lowest number of births recorded since 1946, caused by a slight increase in deaths and an increase in registry cancellations to emigrate abroad (+8.1%). Furthermore, if migratory flows previously mitigated this shortage, today the foreign population’s growth rate is decreasing (−8.6%). Low fertility relates to the concept of the “replacement rate,” which is the natural replacement capacity of the population. If the reproductive rate equals 1, a woman will only replace herself. Still, to maintain constant demographic growth, it is necessary to maintain a balance between birth and mortality. For this reason, the number of births must be higher than that of deaths. Considering a slightly higher number of male children, a suitable replacement rate occurs only when the number of children is at least equal to 2.1 for each woman (Corchia, 2016). This data does not apply to most Mediterranean countries, such as Italy. Indeed, in 2021 the Italian replacement ratio was equal to 1.25, highlighting the imbalance between births and deaths (ISTAT, 2022a). Italy has already experienced periods of prolonged demographic decrease in the past. It happened in the post-World War II period between 1947 and 1951; it also occurred in the 1970s, following the “baby boom,” and lasted until 1995. The worst demographic decrease was recorded during this period, followed by an apparent quiet time marked by some signs of recovery. Then in 2008, the economic and financial crisis, in conjunction with other structural components of the population of childbearing age, interrupted the positive trend (Blangiardo, 2020). An analysis of the data issued by ISTAT in recent years reveals a continuous and constant decrease in the population (ISTAT, 2022b). The lowest value of natural replacement deficit between births and deaths since the Unification of Italy (1861) was recorded in 2020. Before this year, the lowest value occurred in 1918 (−648,000), caused by the Spanish epidemic (ISTAT, 2021c). In 2020, alongside complex factors responsible for the population decrease, the Covid-19 pandemic was added, accentuating the demographic recession trend already underway (ISTAT, 2021c). Starting from February 2020, the impact of the Covid-19 pandemic and lockdown restrictions on the population’s reproductive choices must also be considered. A climate of fear and uncertainty was established, causing postponements in couples’ intentions to have children and accelerating the already declining trend in population dynamics since 2008 (Blangiardo, 2021). The effects of the Covid-19 epidemic occurred mainly in November and December 2020 due to conceptions registered during the lockdown period and the early months of the epidemic wave. Thus, the lockdown restrictions affected parenting couples who decided to postpone parenthood. These effects continued into the first months of 2021: the number of children conceived during the lockdown period, born in January and February, dropped further. March saw a reversal of the trend, with a 3.7% increase in births compared to the previous year, thanks to conceptions between the two pandemic waves of 2020. This change can be attributed to a calmer transition phase characterized by the illusory perception that the epidemic emergency was over (ISTAT, 2021a). Unfortunately, however, it turned out to be a positive trend that only lasted for a few months and collapsed between June (−5.9%) and July (−5.8%), about 9 months after the second pandemic wave (ISTAT, 2021b). From August, the contraction of pregnancies seemed less marked; between November and December, the first signs of recovery emerged, recording a consistent number of births (respectively +6.8% and + 13.5%) (ISTAT, 2022a). This trend analysis could suggest that women’s desire for motherhood diminished, following the decline in the birth rate. Nevertheless, according to the available data, the average number of children that parents desire is higher than the fertility rate and exceeds the replacement rate (ISTAT, 2015). Indeed, the ideal number of children an individual would like to have under ideal living conditions (Lutz et al., 2006) equals 2.3. This difference between the desire to have more children and the actual number of children conceived at childbearing age (considered between 19 and 45 years) is called the fertility gap (CHESNAIS, 2000). It could be interpreted as an unmet need for fertility and as women’s uncertainty in their intentions due to dependence on unstable and constantly changing contexts and circumstances.

## 3. 2030 Agenda for sustainable development and low fertility

Looking closely at the 17 Sustainable Development Goals identified in the UN’s 2030 Agenda (2015) is possible to find several goal targets that lead to address low fertility and the fertility gap. Indeed, the fertility gap poses significant challenges to present and future sustainability. In OECD’s Italy Governance Scan for Policy Coherence for Sustainable Development (2021), low fertility is indeed highlighted by most Italian regions and cities as one of the biggest challenges for building a sustainable future, together with climate change. Venetian Association Sustainable Development, Forum di Limena (2019) claims that “Demographic sustainability is a transversal theme to the Sustainable Development Goals of the 2030 Agenda, even if it has not yet fully entered the sustainability strategies at international and national/regional levels.” Moreover, at an international level, addressing the low fertility and fertility gap is also central for European Union politics for sustainability, as testified by the publication in 2021 of a Green Paper on the topic (European Commission, 2021). These themes are analyzed through the lens of sustainability and well-being throughout the whole life cycle. This leads to the identification of 3 areas of intervention, in the training, working and retirement years, also considering the emerging needs deriving from a constantly growing number of elderly people. Using this lens of analysis, a clearer nexus appears between the 17 SDGs and the opportunity and necessity of addressing low fertility and fertility gap issues in a lifelong, Life Design-driven (Savickas et al., 2009) perspective. Among the others, this contribution highlights the close and mutual relationship between addressing the fertility gap and: (a) Goal 5, “Gender Equality”; (b) Goal 8, “Decent Work and Economic Growth.” (a) The persistent social and cultural problem of unpaid domestic work, as well as the inequitable and restricted access to sexual and reproductive health, are closely related to a decrease in parenthood planning and mostly strike women worldwide. Target 5.4 (recognizing and valuing unpaid care work and domestic work, promoting shared responsibility within the household) and 5.6 (ensuring universal access to sexual and reproductive health and reproductive rights) are specifically dedicated to this. (b) A demanding work-life balance, as long as the absence of correct job retribution hinders people’s ability to plan and enact parenthood. The perceived difficulty of making ends meet and finding time for yourself makes it more difficult to even think about having children. Target 8.5 (achieve decent work for all women and men, including for young people and people with disabilities, and equal pay for work of equal value) addresses this issue.

## 4. Life design paradigm

Therefore, in such a scenario, in which addressing the fertility gap and low fertility demand the parallel consideration of multiple social and economic factors of complexity, it may be helpful to refer to the Life Design paradigm (Savickas et al., 2009). This approach was born to respond to the rapid social and cultural changes and crises that have strongly impacted people’s lives, choices, and economic and social possibilities in the 21st century. Nevertheless, Life Design Career Guidance supports people facing complexity, fostering their ability to adapt and be ready to anticipate and face change and unpredictability (Savickas, 2012). Life Design focuses on developing transversal skills and resources for constructing life and career trajectories in line with personal desires, satisfaction, and well-being. This paradigm is proposed as an essential element of protection and prevention from inequality dynamics, as a means to foster the acquisition of awareness of one’s own resources and skills in order to create one’s own path facing the world’s complexities and challenges. Developing these resources can be fostered through specific training proposed by the paradigm. Therefore, career guidance interventions in the Life Design perspective start from reconstructing individuals’ personal stories and experiences. This process contributes to constructing a more hopeful and optimistic vision, considering reality’s constraints and acknowledging the resources needed to face as well as deconstructing the irrational and stereotypical ones. To do so, Life Design interventions aim at developing people’s skills to resist, persevere and adapt to a complex world (Guichard, 2018). This framework is therefore structured to be life-long, holistic, contextual, and preventive (Savickas, 2012). It is emphasized that the Life Design paradigm adapts to people of all ages and life stages, suggesting a way of valuing needs, desires, and skills to make more effective choices. In line with this, in the Life Design perspective, particular emphasis is placed on critical dimensions useful for guiding one’s life design, especially in crucial moments of change, such as changing or finding a job and transitioning to parenthood, facing the related challenges they may present. These dimensions are career adaptability, optimism, hope, life satisfaction, and resilience (Ginevra et al., 2018, 2020). From a Life Design perspective, values and skills constitute the guide and tool to deal with change and adaptation to new needs and integrally involve the individual, calling him/her to use cognitive, affective, and behavioral components. Moreover, these dimensions allow for better adaptation in life’s most essential decision-making phases. Their development can be effective and functional in the re-construction and co-construction of people’s personal and/or professional trajectories (Savickas, 2012). In line with the Life Design Approach, the aim of this paper is to put women and couples back at the center of life choices, building decent and equitable paths and projects for all.

## 5. Goal of the study

Starting from the previously analyzed literature, it is evident that women’s attitude towards motherhood can no longer be the only aspect to explain the low fertility and fertility gap. It is increasingly essential to investigate other factors. Indeed, according to Bachrach and Morgan (2013), to better understand the reasons for the fertility gap is crucial to investigate the relationship with the economic and social factors of a country. Such as it is vital to consider both competing goals (education, work, and leisure) and adverse circumstances (unemployment rate; Bongaarts, 2001). Moreover, while motherhood attitudes have been frequently studied, some areas of the fertility gap still need to be better understood, and additional research is required. For instance, considering the Italian context, the last data and report about the ideal number of children are from 2015. For this reason, based on the Life Design approach for social and gender equity, the general goal is to analyze the psychological and social motivations for the fertility gap, exploring the difficulties of parenthood planning in a context characterized by gender discrimination and social-economic uncertainty. In particular, considering the literature presented and the necessity to update data and to enlarge the perspective on the fertility gap, the specific goals will be (a) to observe if the differences between the ideal number of children and the number of children actually expected still exist in Italian women; (b) to observe at which age women want the first child; (c) to intercept which aspects can influence the parenthood planning process, focusing on the perception of personal values (internal barriers), and social uncertainties (external barriers); (d) to analyze the relationship between the process to plan a child and the perception of gender stereotype and the motherhood pressure on women.

## 6. Materials and methods

### 6.1. Participants

Participants in the study were 835 Italian women. Three hundred and twenty-four participants (4%) were below 24 years old, and 288 participants (34.5%) were between 25 and 29 years old. The second largest range is from 30 to 34 years old, with 130 participants (15.6%). From 35 to 39 years, there are 51 participants (6.1%); from 40 to 44 years old, there are 14 (1.7%), and finally, from 50 to 54 and from 55 to 59 years old, we find, respectively, 7 and 3 participants who together, correspond to 1% of the sample. Concerning schooling levels, 20 have a secondary school leaving examination (2.4%), 332 have a high school leaving examination (39.8%), 241 have a first degree (28.9%), 169 have a master’s degree (20.2%), 43 have a postgraduate specialized master’s (5.1%), 15 a Ph.D. (1.8%) and 15 subjects selected the “other” option (1.8%). Most of the women (630 participants - 75%) declare themselves unmarried, 96 married (11.5%), 13 divorced (1.6%), 94 select “other” relationship (11.1%), only one is widowed, and two participants prefer not to answer this question. Four hundred and seventy-six women (57%) are employed, while 359 (43%) are not.

### 6.2. Procedure

A snowball sampling procedure (Goodman, 1961) was used to engage the adult women, sharing the questionnaire multimedia link with them. In particular, most of the women were recruited by disseminating the link *via* the social network Instagram, thanks to two public profiles that made the questionnaire link visible and accessible. Before starting, all the participants read the explanation of the project’s goals, the privacy information, and the professional confidentiality declaration. All the participants gave informed consent. The study was conducted following the ethical procedures of the Italian Society for Vocational Guidance (SIO), in line with the “Declaration of Helsinki” and the Oviedo Convention. In particular, the research was approved by the Ethical Committee of Milano-Bicocca University. All participants were obliged to answer all the items. The entire questionnaire lasted approximately 30 min.

### 6.3. Measures

#### 6.3.1. Attitudes toward women and motherhood scale

This scale (Holton et al., 2009) aims to measure attitudes about women’s roles, responsibilities, and expectations related to motherhood. It consists of 18 items for three factors. The first factor refers to the vision of motherhood as normative; an example of an item is “It is selfish not to have kids.” The second factor concerns women’s roles and responsibilities, such as “A high level of education is more important for a man than a woman.” The last factor measures beliefs regarding motherhood in the Italian context, such as “In Italy, women are seen more favorably if they have children.” Each item is rated on a five-point Likert scale, ranging from strongly agree (1) to strongly disagree (5). The subscale of particular interest in the present study is the third one, regarding beliefs about motherhood in the Italian context. In the present study, Cronbach’s alpha for the last scale corresponds to 0.70.

#### 6.3.2. Career barriers inventory

The original scale (Swanson et al., 1996) consists of a 5-Likert scale of 70 items divided into thirteen scales investigating potential barriers people may encounter in their careers. In the questionnaire examined, a reduction of the CBI scale was used, with the same factor structure but composed of 39 items that explore the following barriers:

- Sex discrimination, a scale initially composed of seven items addressing different aspects of discrimination, including economic barriers in the workplace (e.g., “Being paid less than colleagues of the opposite sex from me”)
- Multiple role conflict, assessed by eight items (e.g., “Experiencing stress at work that could affect family life”)
- Conflict between children and work demands, scale initially composed of seven items (e.g., “Feeling guilty about going to work when the children are young”)
- Disapproval by significant others consists of three items on disapproval caused by one’s job and career choice (e.g., “Having a partner who does not approve of my career choice”)
- Discouragement from choosing non-stereotypical careers, composed of five items (e.g., “Feeling discouraged from undertaking professional activities that are traditionally not considered appropriate for one’s gender e.g., mechanical professions for women, educational professions for men”)
- Constraints of the labor market, four items (e.g., “Recognize that there are few possibilities of job placement in the professional sector for which you have trained”)
- Difficulty with networking or socializing, consisting of five items (e.g., “Not knowing the ‘right people’ to advance in one’s profession”).

Considering the goal to investigate the influence of gender stereotypes, the subscale of particular interest in the present study is “Discouragement from choosing non-stereotypical careers.” For this sample, Cronbach’s alpha for this scale is 0.78.

#### 6.3.3. Portrait values questionnaire – PVQ

The PVQ (Schwartz et al., 2001) is a scale composed of 40 items, which indirectly measure the participants’ values through judgments of similarity with another person concerning goals and aspirations. The scale is a 5 points Likert scale ranging from 1 (“very similar to myself”) to 6 (“not at all similar to myself “). The ten types of values identified by Schwartz are (a) *Power*: social status and prestige, control or dominion over people and resources (e.g., “He likes to be in charge and tell others what to do. He wants people to do what he says.”); (b) *Achievement*: personal success through demonstration of competence according to social standards (e.g., “Being very successful is important to him. He likes to stand out and to impress other people.”); (c) *Hedonism*: personal pleasure and sense of gratification for oneself (e.g., “He really wants to enjoy life. Having a good time is very important to him.”); (d) *Stimulation*: Excitement, novelty, and challenge in life (e.g., “He looks for adventures and likes to take risks. He wants to have an exciting life.”); (e) *Self-direction*: independent thought and action - choose, create, explore (e.g., “He thinks it’s important to be interested in things. He is curious and tries to understand everything.”); (f) *Universalism*: understanding, appreciation, tolerance, and protection for the well-being of all people and nature (e.g., “He thinks it is important that every person in the world should be treated equally. He wants justice for everybody, even for people he does not know.”); (g) *Benevolence*: preservation and improvement of the well-being of people with whom one is in frequent personal contact (e.g., “He always wants to help the people who are close to him. It’s very important to him to care for the people he knows and likes.”); (h) *Tradition*: respect, commitment, and acceptance of the customs and ideas that traditional culture or religion provides to the self (e.g., “He thinks it is important to do things the way he learned from his family. He wants to follow their customs and traditions.”); (i) *Conformity*: containment of actions, inclinations, and impulses that might upset or harm others and violate social expectations or norms (e.g., “He believes that people should do what they are told. He thinks people should follow rules at all times, even when no one is watching.”); (j) *Security*: safety, harmony, and stability of society, relationships, and oneself (e.g., “The safety of his country is very important to him. He wants his country to be safe from its enemies.”).

In the present study, Cronbach’s alpha for the scales are: Power: 0.75; Achievement: 0.80; Hedonism: 0.69; Stimulation: 0.68; Self-direction: 0.62; Universalism: 0.66; Benevolence: 0.76; Tradition: 0.64; Conformity: 0.62; and Security: 0.64.

In addition to using the scientifically validated scales reported above, some open questions were formulated in line with the research goals. The topics explored are as follows:

- Thinking about your future, how many children would you realistically want to have?
- Thinking about your future, how many children would you like to have if you had a magic wand? Could you explain the reason for this choice?
- At what age would you like to have your first child? Could you explain the reason for this choice?

## 7. Data analysis

As suggested by Leech and Onwuegbuzie (2009), a mixed-methods design was chosen to achieve the different goals of the study. A quantitative procedure was used to test the relationship between the number of future child planning, personal values, and the perception of gender stereotypes. Quantitative data analyses were performed using IBM SPSS Statistics (Version 28). Secondly, the qualitative analysis was considered more appropriate to explore women’s motivations for planning or not to start a family, with a paper/pencil method. First, all study variables’ means, standard deviations, and Pearson correlations were carried out. Then, one hierarchical multiple regression analysis (Stone-Romero and Anderson, 1994) was carried out to determine the effect of women’s socio-demographic variables (age, schooling, and marital status), personal values (Power, Achievement, Hedonism, Stimulation, Self-direction, Universalism, Benevolence, Tradition, Conformity, Security), and stereotypical factors (Beliefs regarding motherhood in the Italian context and Discouragement from choosing non-stereotypical careers) on the number of realistically planned children. For the hierarchical multiple regression analyses, in step 1 of the regression model, women’s socio-demographic variables were entered. In step 2, personal values were added. Finally, the stereotypical factors were entered in step 3. The “thematic decomposition analysis” (Ussher and Mooney-Somers, 2000) was used for the qualitative data analysis. This approach considers language as constitutive of meanings and the construction of meanings as the outcome of social processes. It is based on the research of themes emerging from the data. According to Braun and Clarke (2006) procedural indications, the coding labels were assigned after familiarization with the data. These were then transformed into ‘thematic categories’ by identifying the relationships between the different codes. This phase was followed by the return to the original transcripts, according to: “a constant moving back and forward the entire data set, the coded extracts of data that you are analyzing, and the analysis of the data that you are producing” (Braun and Clarke 2006, page 15).

**Table 1:** Frequencies of women that realistically and ideally plan child/children.

Number of children	Frequency of women that realistically planned	Percentage of women that realistically planned	Frequency of women that ideally wanted	Percentage of women that ideally wanted
0 Child	260	31.1%	168	20.1%
1 Child	95	11.4%	63	7.5%
2 Children	323	38.7%	307	36,8%
3 Children	142	17.0%	210	25.1%
4 Children	13	1.6%	56	6.7%
5 Children	1	0.1%	16	1.9%
More than 5 children	1	0.1%	15	1.8%

## 8. Results

Regarding our goal a, the following table shows the number of children women realistically plan and the ideal number of children they would like (Table 1). In addition, addressing our goal b, the age at which they want children was analyzed. Considering women that want at least one child, only 1% want them before 25 years old; 21.8% want them between 26 and 30 years old, the 38% want some children between 31 and 35 years old. From 36 years old to 40 years old, 7.2% of women want a child, and only 0.9% want a child after 41 years old. Correlations for all the variables are in Table 2.

**Table 2:** Correlations among the number of children women realistically planned, values, and stereotypical factors.

Measure	1	2	3	4	5	6	7	8	9	10	11	12	13
1. Number of children women realistically planned	1	0.007	0.023	−0.010	0.019	−0.087*	0.095*	−0.047*	0.169**	0.132**	0.074*	−0.120**	−0.088*
2. Power		1	0.657**	0.182**	0.359**	0.290**	−0.045	−0.025	−0.032	0.092**	0.229**	0.018	0.065
3. Achievement			1	0.237**	0.380**	0.352**	0.143**	0.138**	−0.027	0.200**	0.308**	0.059	0.062
4. Hedonism				1	0.262**	0.374**	0.220**	0.215**	0.063	0.094**	0.142**	−0.012	0.025
5. Stimulation					1	0.460**	0.230**	0.244**	−0.015	−0.043	0.089*	−0.017	0.118**
6. Self-direction						1	0.279**	0.395**	−0.043	0.002	0.196**	0.131**	0.081*
7. Universalism							1	0.455**	0.241**	0.266**	0.189**	−0.018	0.069*
8. Benevolence								1	0.092**	0.173**	0.308**	0.126**	0.085*
9. Tradition									1	0.476**	0.294**	−0.205**	0.160**
10. Conformity										1	0.428	−0.067	0.087*
11. Security											1	0.021	0.173**
12. Beliefs regarding motherhood in the Italian context												1	0.022
13. Discouragement from choosing non-stereotypical careers													1

*p < 0.05; **p < 0.01.

Considering our goal c from a quantitative perspective, the hierarchical multiple regression showed that Model 1 with women’s socio-anagraphic variables was significant (∆*R*^2^ = 0.076, *p* ≤ 0.001). Specifically, only the variable “age” negatively influences the number of realistically planned children, as reported in Table 3. All the other socio-anagraphic variables were found to be not significant. Moreover, about our goal c, model 2 with the women’s personal values was significant (∆*R*^2^ = 0.133 <0.001). Specifically, the value of Tradition and Universalism positively predicted the number of realistically planned children. On the other hand, the value of Self-direction and Benevolence negatively predicted the number of realistically planned children. All the other values were found to be not significant. Regarding our last goal (goal d), Model 3 testing the perception of gender stereotypes was significant (∆*R*^2^ = 0.146, *p* ≤ 0.05). Specifically, both Beliefs regarding motherhood in the Italian context and Discouragement from choosing non-stereotypical careers negatively predicted the number of realistically planned children.

**Table 3:** Hierarchical Multiple Regression analyses predicting the number of realistically planned children.

Predictor	∆*R*^2^β
Step 1	0.076**
Women’s age	−0.286**
School grade	0.034
Marital status	0.250
Step 2	0.133**
Power	−0.046
Achievement	0.052
Hedonism	0.043
Stimulation	−0.049
Self-direction	−0.143*
Universalism	0.133*
Benevolence	−0.172*
Tradition	0.186*
Conformity	0.041
Security	0.073
Step 3	0.146*
Beliefs regarding motherhood in the Italian context	0.028*
Discouragement from choosing non-stereotypical careers	0.054*

**p < 0.001; *p < 0.05.

Analyzing the qualitative data, to get deeper and multimethodological insight about our goals c and d, the motivation for having zero or at least one child was examined. In Table 4, it is possible to see all the categories. These categories are considered non-exclusive, as the same participant in his answer could refer to different motivations. Several women cannot really explain the reason for their parenthood planning (18.3% answers), and some women recognize personal reasons (21.4% answers) as the most important for having zero or one child. In this category, it is possible to consider, “Women do not like children (2.7% answers)” and the fear connected to the physical experience related to childbirth (3.1% answers about fear of the labor and 2.4% answers about the changes in the body). Several women do not want any child because they do not consider themselves good enough (9.5% answers), especially considering the enormous responsibility that a child implicates (3.7% answers). Most women refer to some social and contextual motivation (51.3% answers). First of all, the absence of the State, that does not support and does not offer services to help women to consider this choice (7.8% answers). In this context, women report having to choose between personal freedom or the freedom to have a career (9.2% answers) or having a child. In addition, some of women communicate that they – or their couple - do not have enough money to raise a child (13.6% answers), especially considering a perception of economic certainty. Moreover, some of them (13.6%) describe several phenomena connected with the specifically female condition: work-life balance, stereotypes, discrimination, etc. The perception of this external barrier is considered a high hurdle to the decision to have a child. Finally, some women (7.1% answers) report as relevant the characteristics of the global context: overpopulation, inequality, and climate change.

**Table 4:** Type of motivation to not have a child or not to have more than one.

Categories	Examples	Frequency
I do not want them	“I never wanted them. I do not care to have them” (N. 347)	18.3%
	“I do not want any children” (N. 322)	
Do not like children	“I do not like children” (N. 52)	2.7%
	“I do not like children, and I do not have a maternal sense” (N. 635)	
Fear of the deliver	“Because I do not want to give birth” (N. 525)	3.1%
	“Fear of labor.” (N. 186)	
The changes in the body	“It is a physical discomfort” (N. 425)	2.4%
	“I am afraid of my body changing” (N. 373)	
Not feeling to be a good mother/good enough	“I do not feel capable of raising and educating a child”	9.5%
	“I love children, but I do not think I would be able to raise one” (N. 123)	
Too much responsibility	“Too much responsibility and commitment” (N. 107)	3.7%
	“Responsibility”(N. 822)	
A generic absence of the State	“I do not have the necessary means and the necessary support from the state” (N. 678)	7.8%
	“I do not live in a country that would allow me to balance family and career” (N. 503)	
Loss of freedom	“Because I am a bit selfish and I want more time for myself” (N.726)	9.2%
	“I still want to have the freedom that people without children have” (N. 507)	
Lack of economic security	“Because I like children, but you need money to raise one” (N. 85)	13.6%
	“I do not think I can afford to support economically more than one child” (N. 48)	
	“There is no money” (N. 380)	
Female condition	“Considering the socio-economic context of the country and the condition of women, a woman is even more convinced that she does not want them” (N. 94)	13.6%
	“I think that being a woman, I could not decide to have a career and be a mother at the same time. If I were a man, I would have a different opinion” (N. 440)	
	“Work-Life Balance!” (N. 752)	
	“Gender Stereotypes” (N. 195)	
Environmental, and ethical choice	“I would rather adopt because we are already on an overpopulated and exhausted planet. (N. 70)”	7.1%
	“They would have a horrible life due to the climate crisis and the collapse of ecosystems (N. 77)”	
Other	“I do not know” (N. 325)	9.2%
	“Why yes?” (N. 408)	
	“/” (N. 235)	

## 9. Discussion

This paper considers the process of the fertility gap and birth rate from a psychosocial perspective. Therefore, it highlighted the relevance of the Life Design approach to better comprehend the factors involved in these issues and foster sustainable and equal personal and parental planning for women and men. The context of reference for the analysis proposed is the Italian one, that like and more than others, is marked by gender asymmetries and disparities in all spheres (private, public, personal, family, professional, social, political) (Eurostat, 2020; OECD, 2021; ISTAT, 2021a,b,c). The permanence of gender stereotypes in the female and male population, the presence of gender violence, the low presence of women in the labor market, the lack of social infrastructure and personal services, and the persistence of a family welfare model, which offloads the reconciliation of lifetimes onto women, give rise to various consequences. These consequences include the voluntary renunciation of paid work, as well as a constant difference between the number of children desired and those had. As shown by this study’s results, according to our goal a (to observe if the differences between the ideal number of children and the number of children actually expected still exist in Italian women), it is possible to confirm Italian women’s desire to have at least 2 children (ISTAT, 2015) which is not met, often because of bad contextual social and economic conditions. Added to this is the growing poverty of women and the persistence in Italy and all over the world of organizational models that reward availability “without time limits.” It is hence possible to observe, especially in highly educated and STEM fields, the strengthening of “all time-consuming” professions, which seem unlikely to include possibilities for a sustainable “work-life balance” (Camussi et al., 2022). Faced with this situation of barriers and enduring inequality, an important part of the adult female population is comprehensibly reacting with an attitude of resigned, passive acceptance of reality as it is. As shown by this study, women are therefore not willing to give birth to children in a world that is making them struggle socially and economically (as we inquired according to our goal c, to intercept internal and external barriers influencing parenthood planning process). Moreover, from a psychological point of view, it is not unusual that those who experience greater disadvantage in the social context are the ones ending up believing the reality they experience to be just and acceptable. It would be too painful and destabilizing to simultaneously recognize the world both as unfair and extremely resistant to change (Camussi et al., 2021). Therefore, after taking into consideration these complex factors and their implications, in line with 2030 Agenda Goals 5 and 8, one of the main aims must be to identify and remove the obstacles that do not allow women and men to realize their parenthood planning. As highlighted by this study’s results, considering our goals c (internal and external barriers vs. parenthood planning) and d (gender stereotypes and parenthood pressure vs. parenthood planning) implementing interventions involving the multiple levels that compose the complex challenge of the fertility gap would not only benefit birth rates (Nie et al., 2019). Given that both gender equity and decent work themes are to be considered, it would create the conditions to increase individual and community well-being as long as social sustainability. Therefore, as the results of this study show, it would be useful to focus on the issue of gender equity, which still negatively influences women’s satisfaction and their parenthood planning (goal c and d). Actions that can be implemented to promote gender equity may consist of encouraging practices that support the enhancement of women’s potential and resources, as well as men’s. At the same time, through preventive career guidance actions, it is necessary to implement the personal and social resources of individuals and couples, so that they can support people in the processes of re-construction and co-construction of their personal trajectories. The goal of these interventions should therefore be to bring women and couples back to the center of their life choices, building decent, viable and equitable pathways and projects for all. In line with scientific literature, this work shows that adopting a Life Design approach can help individuals navigate the challenges of sustainable parenthood by fostering intentional decision-making and purposeful action in various domains of life. By integrating sustainability principles and practices into their life design, individuals can contribute to a more sustainable and just future for their children and future generations (Greco et al., 2022). Life design interventions can help women and men balance the demands of sustainable parenthood and personal well-being (Camussi et al., 2021). For instance, to match the fertility gap, Lappegård (2020) proposed to intervene in adults by developing adaptive coping strategies to balance the competing demands of work, family, and sustainability goals, as well as addressing gender stereotypes. In fact, Suero (2023) found an inverse relation between Spanish women willing to have a second child and a stereotypical gendered division of housework. There is, therefore, a need to implement interventions aimed at reducing the impact of barriers (external and internal) on women’s life design and motherhood planning and at the same time able to raise society’s awareness on social issues, towards real cultural change. Young and older women should be supported to develop resources and seek social support that can enable them to achieve their goals. Therefore, it would be useful to promote social awareness about gender barriers and changes in the living and working environment, building viable solutions through the involvement of the entire community of experts and citizens. In conclusion, after considering all the multiple factors involved in the widespread phenomenon of low fertility, it is necessary to remove the existing obstacles to allow men and women to fulfill their family and life plans. Therefore, it is necessary both nationally and internationally to implement policies to support the birth rate, offering to parental couples guarantees on several fronts. The starting point should be the knowledge of the phenomenon to raise awareness among the population, including interventions on cultural factors and actions to support families to facilitate work-life balance. Faced with this extremely complex context, this research can inspire and positively influence possible new interventions to be implemented to improve quality of life and promote well-being and sustainable life planning of a single category of people and the entire community.

## 10. Limitations and future research perspective

Although some interesting results emerged, this study presents some limitations. One possible limitation of the research may relate to the self-report questionnaire. It may be unreliable and subject to systematic biases such as deception and social desirability. Indeed, despite the anonymity, participants may have responded according to what society considers acceptable and appropriate, especially given the high sensitivity of the topic. Concerning the use of open-ended questions, some limitations can also be noted in the qualitative section of the research. People tend to show only some aspects of themselves, while interviews or closed questions allow in-depth explorations. Starting from these limits, future directions of research can be identified. Firstly, the research could be expanded to be more consistent and representative of all genders and the Italian population. Furthermore, controlling the geographical belonging, possible variations in results between different geographical areas characterized by dissimilar socio-economic developments and welfare policies could be analyzed. In addition, the research could also be extended to a male sample; this would make the sample more balanced and representative, also allowing a comparison between genders. Finally, future research could be accompanied by semi-structured interviews to further explore specific topics and capture even the most implicit aspects of the responses.

## Data availability statement

The raw data supporting the conclusions of this article will be made available by the authors, without undue reservation.

## Ethics statement

The studies involving human participants were reviewed and approved by Research Commission of the University of Milano Bicocca. The patients/participants provided their written informed consent to participate in this study.

## Author contributions

ECam: coordination (lead), conceptualization (equal), and review and editing (supporting). DM: conceptualization (equal), writing – original draft (equal), and writing – review, and editing (supporting). RR: writing – original draft (equal) and writing – review and editing (equal). MS methodology and formal analysis (supporting), writing – original draft (equal), and writing – review and editing (supporting). ECal: writing – original draft (supporting) and writing – review, and editing (supporting). CS: writing – original draft (supporting) and review and editing (supporting). CA: conceptualization (lead), writing – original draft (lead), methodology and formal analysis (lead), and writing – review, and editing (equal). All authors contributed to the article and approved the submitted version.

## Conflict of interest

The authors declare that the research was conducted in the absence of any commercial or financial relationships that could be construed as a potential conflict of interest.

## Publisher’s note

All claims expressed in this article are solely those of the authors and do not necessarily represent those of their affiliated organizations, or those of the publisher, the editors and the reviewers. Any product that may be evaluated in this article, or claim that may be made by its manufacturer, is not guaranteed or endorsed by the publisher.
